# After-Results of Treatment of Caries by Geranium-Formol

**Published:** 1901-01

**Authors:** C. André, G. De Marion


### Abstracts and Translations.

### AFTER-RESULTS OF TREATMENT OF CARIES BY GERANIUM-FORMOL.[1]

BY MM. C. ANDRÉ AND G. DE MARION.

In presenting the results of treating caries of the third and fourth degrees by geranium-formol, a method we introduced several years ago, we have a double purpose. First, to show the success of the method when properly carried out; secondly, to reduce to their real value proceedings which it has been desired to connect with the formol method, and which show that their authors have a complete misunderstanding of the useful properties of this substance for the purpose we are dealing with.

This question of the useful properties of formol is one of great importance, and we feel ourselves bound to accurately determine them. In order to do so let us consider the problem at the commencement, and see what is the condition of a tooth affected with caries of the fourth degree. The pulp has been destroyed and liquefied by putrid fermentation; in its place we find the products of its destruction, and among these products a quantity of infectious germs. The condition is much the same as when animal matter is destroyed in contact with the air; the canals are filled with a brown substance of soft consistence, moisture, emulsionized fatty acids, sulphuretted and phosphorized ammoniacal derivatives, and these, especially the latter, which are soluble in water, are disseminated in the dentinal tubes.

Now, when formol is brought in contact with putrid products there results this remarkable fact of the almost instantaneous deodorization of these residues if the formol has been used in sufficient strength. This important property has been observed and noted by all those who have used formol in treating the dental canals, as well as by surgeons who have employed weaker solutions for washing infected wounds; but we were the first to give a

---

[1] Translated from L'Odontologie.

rational explanation of these facts founded upon the reciprocal chemical action of formol and of ammoniacal products.[1] We may repeat in a few words the facts which serve as a basis for this theory.

When equal volumes of formal and ammonia are mixed together much heat is evolved, and the alkaline odor disappears. The two bodies combine thus: 6 molecules of formol + 4 molecules of ammonia = 1 molecule of hexamethylenamine + 6 molecules of water. The reaction is rapidly effected, and we are sure that it is complete at the end of a quarter of an hour. The ammonia is thus replaced by the hexamethylenamine, which is a white powder very soluble in water and in alcohol, non-volatile, neutral, and which is neither an irritant nor a caustic.

If instead of existing free the ammonia be combined with an organic acid, such as acetic, malic, lactic, or citric acid, the same reaction occurs, setting free the acid. For instance, with acetate of ammonia the action may be thus expressed: 6 molecules of formol + 4 molecules of ammonium acetate = 1 molecule of acetate of hexamethylenamine + 3 molecules of acetic acid + 6 molecules of water.

One of us has made use of this reaction in successfully administering spirit of mindererus as an antidote in a case of poisoning by formol.[2]

And if instead of ammonia we have to do with putrid bases, free or combined, the same thing happens; there is always a combination with the formol, a resulting neutralization of the ammonia base, and a distinct transformation into products more condensed, inodorous, non-volatile, and deprived of all irritating or caustic action.

It must be well noted that up to now the question has not been as to the microbicide action of formol; the only effect considered is a purely chemical one of changing volatile and fetid ammoniacal derivatives into more condensed, neutral, fixed, and odorless products of a constitution analogous to hexamethylenamine, although having more complicated formulæ.

This is not all, for the products of disintegration of the pulp

---

[1] Le fermol géranié en thérapeutique dentaire, par G. de Marion et C. André. Compte-rendu du Congrès dentaire de Paris, Octobre, 1897.

[2] Journal de pharmacie et de chemie, July 1, 1899.

are not formed only of ammoniacal derivatives; there are fatty acids besides, arising from the splitting up of albuminous substances and which give that peculiar soft viscous consistence to the contents of the canals; lastly, there are gaseous products, in small quantity, certainly, principally formed of sulphuretted hydrogen and carburetted hydrogen.

Practically speaking, these fatty acids seem to have no distinctive noxious influence, since they are non-volatile and their chemical energy is very feeble; but we think that by the viscous consistence which they render to the pulp residue they can, by obstructing the microscopic opening of the dentinal canals, oppose a barrier to the diffusion of formol and delay its action. What confirms us in this opinion is the much greater rapidity of the disinfecting action of formol since we employed it in an alcoholic solution the same strength as the aqueous ones.

We may recall, indeed, that the solution we have employed since October, 1897, under the name of formol-geranium has the following composition:

| | |
|---|---|
| Formic aldehyde | 40 parts. |
| Essence of geranium, redistilled | 20 parts. |
| Alcohol, 80 degrees | 40 parts. |

That is to say, that our geranium formic liquid has a strength of formic aldehyde as great as that of the commercial formols and contains a fifth of its weight of pure essence of geranium.

Now, alcohol and essence of geranium which separately have a very marked solvent action upon fatty acids co-operate in a very solvent manner by their association in the general act of disinfection in disintegrating and dissolving the viscous stuff which lines the root walls and obstructs the openings of the dentinal canals. Besides, alcohol by its own diffusibility in moist places helps the diffusion of formol in the fluids of the dentine.

There remain the gaseous products and principally sulphuretted hydrogen and formene, upon which our liquid has no chemical action of absorption. But these products are in small quantities, for they are set free as fast as they are formed. Alcohol, however, and the essence of geranium, which in a general way feebly dissolve gaseous bodies, can facilitate their departure by mixing with the fluids of the tooth.

Now that we have seen how the principal constituents of our combination help to produce perfect disinfection of the root walls and the dentine, it remains to speak of their sterilizing action.

When the destruction of the putrid products is obtained, and only at this moment, the antiseptic work begins. The formol, the diffusion of which is very rapid in the conditions of the spot where it is placed, and the essence of geranium itself, helped by the alcohol, penetrate into the canalicules and destroy all the pathogenic germs. We will not insist upon these questions of diffusibility, no more than upon the considerable antiseptic powers of formol and essence of geranium, antiseptic powers much superior to the necessity caused by the germs. We have established elsewhere these important points, and they are too well known now to require repetition.

The time has come to say something of the methods to which we alluded above.

If one well imbued with this idea that the antiseptic action of formol is subordinate and subsequent to its disinfectant action, that the one can only happen when the other is achieved; if, moreover, one recollects that the disinfecting effect of formol is (according to the reactions we have stated) proportionate to the quantity used, it becomes unnecessary to use any other argument to justify the use of a large dose of formol in dental dressings. We must then repudiate every formulæ in which a weak dose of formol appears under the vain underestimated excuse of its great antiseptic power; as for us, we have given to our solution its minimum strength.

After weak solutions of formol there is another form under which it has been desired to use it in dental therapeutics. We wish to refer to powders or pastes containing formol in a nascent state (?).

We have analyzed a preparation of this kind; it contained oxide of zinc, burnt alum, anhydrous sulphate of lime, eugenol, and an infinitesimal quantity of trioxymethylene. According to the instructions which accompanied it. this powder should be mixed into a paste with a liquid which was glycerin, and used as a unique application to make the most complete filling possible in a cavity under a permanent stopping. Experience has shown a short time afterwards the inadequacy of this mode of treatment.

We do not know if the owners of this powder attribute its virtues to formol; for our part we are sure that it does not intervene, because of its insignificant proportion and its immediate absorption by the putrid products largely in excess. It must, however, be recognized that there was a relative success, and that for some time it caused an arrest in the progress of caries.

The explanation of this fact seems easy to us when we remember the dehydrating qualities of sulphate of lime and burnt alum. It probably happens that these powders absorb by degrees the water contained in the dentine, and as putrid fermentation requires the presence of water, there was arrest of this fermentation and a relative cessation of the morbid phenomena. But there was only an arrest and not the destruction of germs nor disinfection, and when, by a mechanism that we do not inquire into the dehydrating properties of the powders were satisfied and moisture reappeared in the dentine, fermentation recommenced and with it troublesome symptoms.

Nothing like this occurs with our method applied in the way we have several times described already, and which we have by successive steps brought to perfection. Thus, whilst at first we were obliged to use six or eight dressings for a large tooth deeply infected, we have reduced this figure by fifty per cent. Then the interval between two dressings which we fixed at two days has been brought down to twenty-four hours by the use of alcohol as the vehicle for the formic aldehyde and essence of geranium, and again it may be said that this interval much surpasses the necessary time, and could be reduced by some hours if the necessity of proceeding quickly should occur in practice.

Finally, let us recall what we said in our first communication with regard to the cleaning of canals; this cleaning is not an indispensable condition, and if in consequence of irregular conformation the ends of the roots cannot be reached and cleansed, formol modifies the pulp *débris* in such a way as to render them incapable of producing a later infection.

We have not introduced any modification of our method during the last two years. We may briefly say that it consists in making dressings at intervals of twenty-four hours until the last one taken out shows not the slightest trace of fetor, but on the contrary preserves in absolute purity the smell of the geranium. For these dressings strands dipped in the formol-geranium are introduced into the canals and pulp-chamber; the whole is covered in by gutta-percha.

### CONCLUSIONS.

Let us sum up in a few lines what we have just said and what we have said in the former communications upon the use of formol in dental therapeutics.

Geranium-formol realizes as exactly as is possible the recognized theoretical conditions for the treatment of teeth with dead pulps.

1. It is the most powerful disinfectant known. It destroys the products of pulp fermentation, combining with and neutralizing them. This effect is shown by the complete and definite deodorization of the cavity after two or three dressings.

2. Its antiseptic power is superior to that of sublimate.

3. It is extremely diffusible in moist places. By means of this valuable property it acts not only on the root walls up to the apex, but even in the dentinal canalicules as far as the periphery of the tooth.

4. When geranium-formol is placed experimentally in sufficient quantity among putrid products it deodorizes them instantly. This experience shows that dressings may be made with as short intervals as may be desired, one hour if the need for rapidity occurs in practice; generally these dressings are applied on several consecutive days.

5. Geranium-formol does not in any way injure the hard tissues of the tooth, and does not set up any troublesome condition (periostitis) in the membrane.

6. Geranium-formol shows the advantage of a lasting result as compared with absorbent and drying powders. These only act by causing a more or less perfect desiccation of the tooth, but this desiccation is only temporary, and when the powders become hydrated pulp fermentation recommences with the train of symptoms which it excites.—*British Journal of Dental Science.*

